# Variation of Medicinal Components in a Unique Geographical Accession of Horny Goat Weed *Epimedium sagittatum* Maxim. (Berberidaceae)

**DOI:** 10.3390/molecules171113345

**Published:** 2012-11-08

**Authors:** Qiong Liang, Guoyan Wei, Jianjun Chen, Ying Wang, Hongwen Huang

**Affiliations:** 1 Key Laboratory of Plant Germplasm Enhancement and Specialty Agriculture, Wuhan Botanical Garden, CAS, Wuhan 430074, China; 2 Graduate University of Chinese Academy of Sciences, Beijing 10049, China; 3 Key Laboratory of Plant Resources Conservation and Sustainable Utilization, South China Botanical Garden, Chinese Academy of Sciences, Guangzhou 510650, China

**Keywords:** herbal plant, flavonoids, polysaccharides, Chinese medicine

## Abstract

Herbal *Epimedium* species have been widely in Traditional Chinese Medicine for sexual enhancement, immunity improvement, anticancer and anti-aging treatment, with flavonoids and polysaccharides being the major active components. However, exhaustive depletion of wild sources warrants germplasm evaluation and quality resource exploration. A preliminarily analysis had previously indicated that a specific local geographic accession of *Epimedium sagittatum* found in Luotian (LT) county of Hubei Province (China) had a much higher content of total flavonoids and polysaccharides. In this study, we further investigated the medicinal component variation in the LT type under different light intensities and in different regions by the common-garden experiment. The results indicated a light intensity range of 40–160 μmol/m^2^/s was the most suitable for the synthesis and accumulation of total flavonoids, while polysaccharide accumulation was negatively correlated with the light intensity. Icariin was the component displaying the highest content among flavonoids, and the content of major flavonoid bioactive components was relatively stable in the third year after cultivation. There was significant correlation between the major flavonol glycoside constituents and the geographic location, and Central China followed by Northern China were the highly suitable regions for cultivation of LT type *E. sagittatum*. The results revealed that there was a functional balance between flavonoids and polysaccharides at different developmental stages, and the best harvesting stage should consider the primary contents of interest. This study provides important information on the exploration of quality resources, further breeding approaches and cultivation practices of *E. sagittatum*, and thus the important insights to enhance our understanding of quality control of traditional medicinal plants.

## 1. Introduction

Herbal *Epimedium* plants are important traditional Chinese medicinal perennial herbs. In the Chinese medicinal monographs “*Shennong Ben Cao Jing*” *(Shennong Herbals)* (~1,600) and “*Ben Cao Gang Mu*” (Compendium of Materia Medica) (~1578), herbal Epimedii was recorded as “invigorating sex, strengthening muscles and bones”. More functions such as immunity improvement, anticancer, anti-aging, *etc.* were reported too [1]. Flavonoids are the major active components in the leaves of *Epimedium* plants [1,2], and total flavonoids, particularly icariin, have been defined as its quality indicators in the “*Chinese Pharmacopoeia*”. Recently, many studies have proposed four major flavonoids including icariin, epimedin A, epimedin B and epimedin C to be integrative quality indicators of herbal epimedium [3,4,5]. Meanwhile polysaccharides have been proven as being biological active for antitumor, anti-oxidation and immunity improvement [6].

*E. sagittatum* is one of the five species listed in the “*Chinese Pharmacopoeia*” and it is widely distributed in central, eastern and southern China. As a shade-tolerant species, it is usually found under bushes and in gullies in the natural environment. Its bioactive components, such as flavonoids, vary among different geographic regions [3]. In an extensive field survey and preliminarily analysis, we found that a local geographic accession found in Luotian (LT) County, Hubei Province, China, was significantly different from other populations of *E. sagittatum* in morphology, medicinal components and genetic diversity [7], providing us with a valuable material for further study of the selection and breeding of novel varieties for cultivation of this important medicinal plant. 

Since wild herbal epimedium has been the major source of raw medicinal materials for centuries, current market supplies suffer from exhaustive depletion of wild harvests and increasing demand has further deteriorated this situation. The wild resource conservation, germplasm evaluation, quality resource exploration and large-scale cultivation have become critical for the sustainability of the herbal epimedium and related Chinese medicinal plant industries. The objectives of the present study were to evaluate and investigate the seasonal changes of flavonoids and polysaccharide in the LT epimedium type using common-garden experiments, light-controlled experiments and regional trials. It aims to provide important information on the quality resource exploration of *E. sagittatum* and breeding for commercial cultivation, and important insights to enhance our understanding of quality control of key Chinese medicinal plants.

## 2. Results

### 2.1. Seasonal Variations of Flavonoids and Polysaccharides under Different Light Intensities

**Figure 1:**
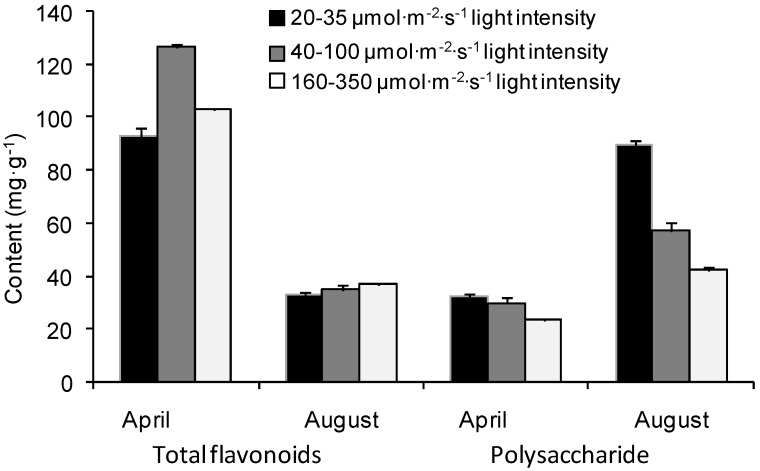
Contents of total flavonoids and polysaccharide influenced by different light intensities in light-controlled indoor cultivation. Bars are standard errors.

Seasonal variations of total flavonoids and polysaccharide content in the light-controlled indoor cultivation experiment indicated that they were highly influenced by light intensity (Figure 1). Polysaccharide content showed a negative correlation with light intensity (Table 1), while there were different influences of light intensity on the accumulation of flavoniods in different growth stages. In April, the content of total flavonoids was the highest under 40–100 μmol/m^2^/s light range and the lowest under 20–35 μmol/m^2^/s, while its synthesis was impeded and the content declined above 160 μmol/m^2^/s. In August, the content of total flavoniods showed a positive correlation with the light intensity (Table 1). In addition, there were significant variations between the contents of total flavonoids and polysaccharide at different growth stages. The contents of total flavonoids in April under different light conditions were 179.9%–264.9% higher than those in August, while polysaccharide contents were 78.7%–178.8% lower than those in August.

**Figure 2:**
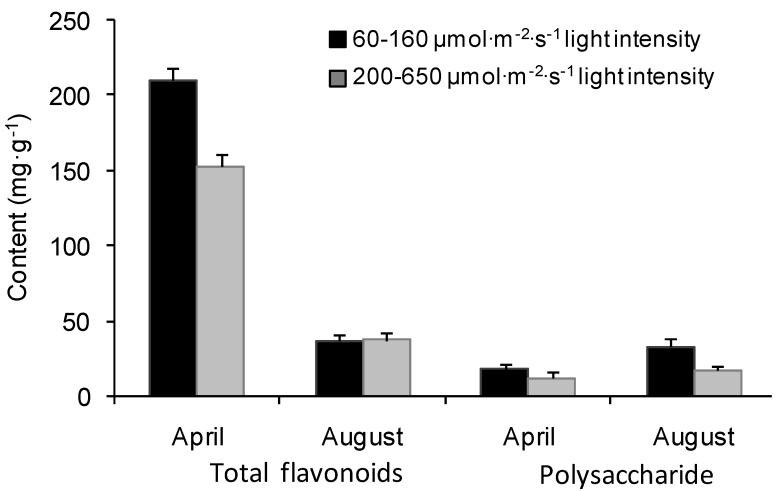
Contents of total flavonoids and polysaccharide influenced by different light intensities in the common garden experiment at Wuhan Botanical Garden. Bars are standard errors.

The common-garden experiment further revealed seasonal variations of total flavonoids and polysaccharide and the effect of light intensity (Table 1; Figure 2). The contents of total flavonoids in April were about five times as much as those in August, while polysaccharide contents in April were 47.6%–87.9% lower than those in August. The content of polysaccharide in the lower light intensity (60–160 μmol/m^2^/s, plot 1) was 54%–96% higher than that in the higher light intensity (200–650 μmol/m^2^/s, plot 2). The total flavonoids was 37% higher in plot 1 in April and 0.8% lower in plot 1 in August than those at plot 2.

**Figure 3:**
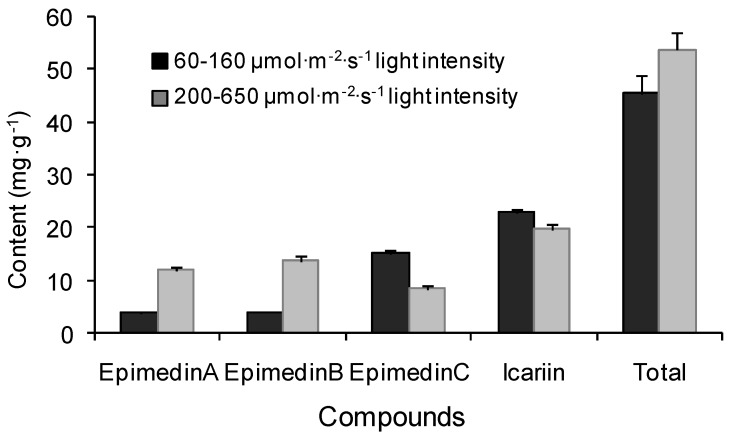
Contents of four flavonoids influenced by different light intensities in the common garden experiment at Wuhan Botanical Garden. Bars are standard errors.

In order to further analyze the influence of light intensity on flavonoids, variations of icariin and epimedin A, B, C in April were investigated in the common-garden experiment (Figure 3).

Icariin had the highest content among the four components in each plot. The content of epimedin A and epimedin B at higher light intensity (200–650 μmol/m^2^/s, plot 2) were 215.7% and 269.2% higher, respectively, than those in lower light intensity (60–160 μmol/m^2^/s, plot 1), whereas the content of epimedin C and icariin were 78.6% and 16.2% lower, respectively. The concentration of the total of four flavonoids was positively correlated to the light intensity (Table 2).

### 2.2. Yearly and Regional Variation of Content of Icariin, Epimedin A, B and C

**Figure 4:**
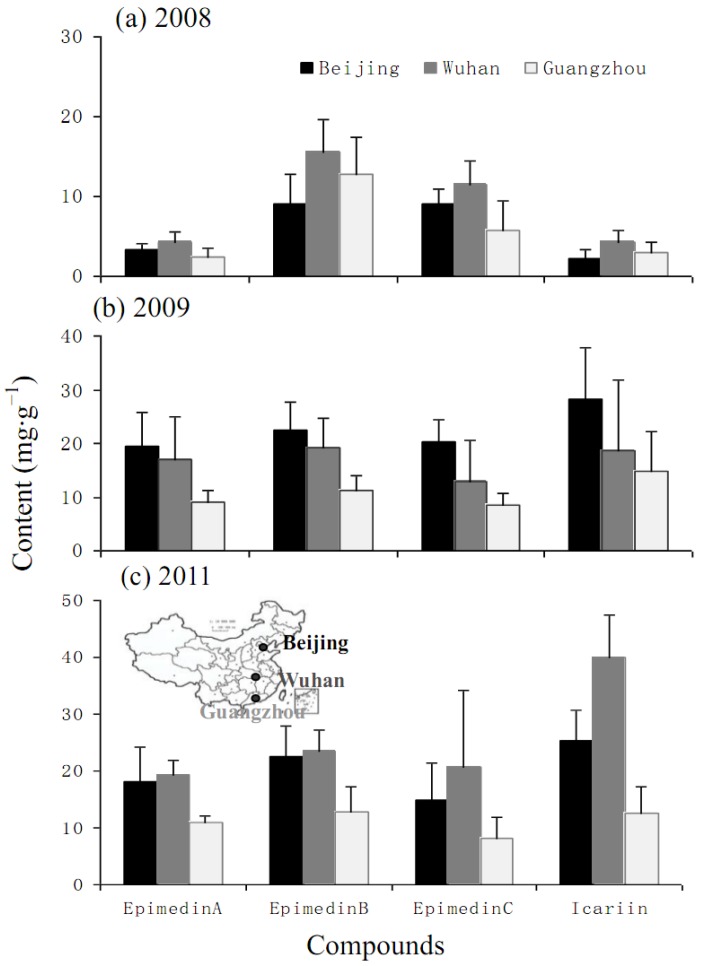
Four flavonoids contents of LT race planted in three gardens (Beijing, light intensity 50–180 μmol/m^2^/s; Wuhan, light intensity 60–160 μmol/m^2^/s; Guangzhou, light intensity 55–170 μmol/m^2^/s) after one (2008), two (2009) and four years (2011). Bars are standard errors.

**Figure 5:**
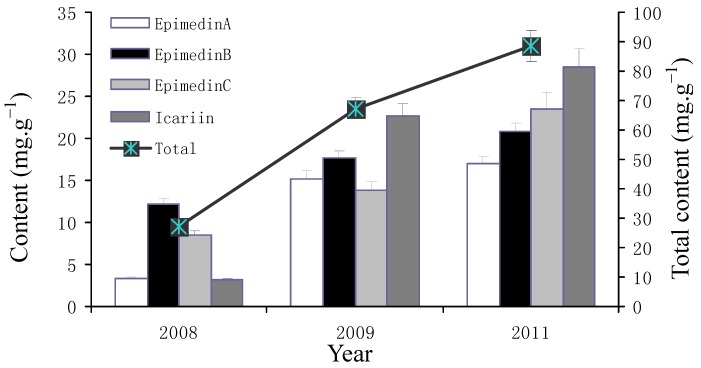
Contents of the four flavonoids in LT race cultivated after one (2008), two (2009) and four years (2011). Values shown are mean for the three gardens (light intensity 50–180 μmol/m^2^/s). The two axes of coordinates represent the content of each flavonoid and the total of the four flavonoids (epimedin A, B, C, and icariin). Bars are standard errors.

**Figure 6:**
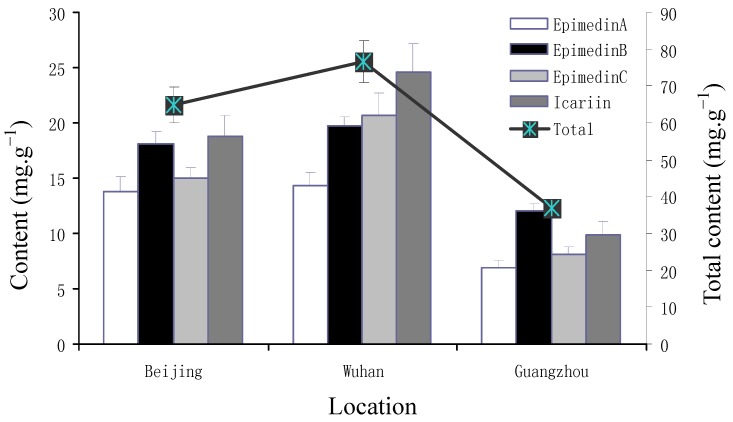
Contents of the four flavonoids in LT race planted in the three gardens (Beijing, light intensity 50–180 μmol/m^2^/s; Wuhan, light intensity 60–160 μmol/m^2^/s; Guangzhou, light intensity 55–170 μmol/m^2^/s). Values shown are mean for contents in the three years. The two axes of coordinates represent the content of each flavonoid and the total of the four flavonoids (epimedin A, B, C, and icariin). Bars are standard errors.

There were significant variations among the contents of icariin, epimedin A, B, C after different duration of cultivation (Table 3; Figure 4). The common garden experiment was set up in October 2007, and the mean content of each main flavonoid in 2008 was the lowest, but increased by 362.8% (epimedin A), 45.3% (epimedin B), 63.7% (epimedin C), and 636.2% (icariin), respectively, in 2009, then stabilized by increasing only 4.8%–11.6% in 2011 (Figure 5). The highest contents of each flavonoid were seen in in Wuhan garden followed by Beijing, while the contents at Guangzhou were only 50.2% (epimedin A), 66.9% (epimedin B), 54.5% (epimedin C), and 52.8% (icariin) of those at Wuhan (Figure 6). Meanwhile, contents of epimedin C and icarrin increased in 2009 and began to decrease in 2011 for plants cultivated at Beijing and Guangzhou, but increased continuously at Wuhan (Figure 4). As far as each flavoniod component was concerned, epimedin B was the highest, with a mean content of 12.4 mg·g^−1^ and icarrin was the lowest with only 3.2 mg·g^−1^ in 2008, while icarrin became the main component with the highest mean content, followed by epimedin B in 2009 and epimedin C in 2011 (Figure 5). Among the different regions, icariin was the main component with the highest mean content both at Wuhan (24.7 mg·g^−1^) and Beijing (18.8 mg·g^−1^), while epimedin B was the highest-content component (12.1 mg·g^−1^) at Guangzhou (Figure 6).

## 3. Discussion

*E. sagittatum* is a shade-tolerant species, and it seems that light intensity plays an important role in biosynthesis and accumulation of flavonoids and polysaccharides. As expected, the polysaccharide content was negatively correlated with light intensity, consistent with its shade-tolerant characteristics [8]. Combining the results of light-controlled experiment and the common-garden experiment (Figure 1 and Figure 2), the lower light intensity of 40–160 μmol/m^2^/s can be recommended for cultivation practice for a high production of total flavonoids and polysaccharides. The result was in disagreement with previous reports on herbal epimedium [9,10] with more contents under intensive light, indicating a difference in response to light intensity among different species. Meanwhile the study showed that there was lower concentration of the total of four major flavonoids, but higher content of total flavonoids, under lower light (Figure 2 and Figure 3) in April, implying that different flavonoids can be induced by different light ranges, probably there were large number of other flavones stimulated in addition to the four major components under lower light. Meanwhile, the contents of epimedin A and epimedin B increased significantly when the light intensity was increased, whereas the contents of epimedin C and icariin obviously declined (Figure 3), indicating that higher light intensity improves the biosynthesis of epimedin A and epimedin B but impedes that of epimedin C and icariin. Further studies are needed to better understand the impacts of regulatory factors on the synthetic balance of the four medicinal flavones in the flavonoid pathway. 

Total flavonoids, especially icariin, are defined as quality indicators of herbal Epimedii, whereas epimedin C was reported much higher than other components in *E. sagittatum*, *E. pubescens*, *E. wushanense*, *E. acuminatum*, and *E. myrianthum* [11,12,13]. Our study showed that icariin was the highest content component (Figure 3 and Figure 6), showing further evidence that the main components vary significantly among different populations or geographical types of *E. sagittatum* and a better utilization value of the LT epimedium accession. Also the content of total flavonoids was high when the content of polysaccharide was low and *vice versa* in different growth stages (Figure 1 and Figure 2), suggesting that there is certain functional synthetic balance between flavonoids and polysaccharides during different growth stages. Coincident conclusions were reported for crop plants, herbal *Epimedium* and *Dioscorea opposite* Thunb [14,15,16], but opposite results, *i.e.*, the lowest content of polysaccharide in fructification stage on *Cynomorium songaricum* Rupr., was also observed [17]. The balance mechanism between flavonoids and polysaccharides needs further investigation.

The *Epimedium* herb is a perennial plant and is usually harvested in autumn in cultivation practice because of its yield, and the contents of main medicinal components (e.g., flavonoids, polysaccharides), are also the main factors used to decide the time of harvest. Previous research on the best harvest time were mainly concentrated on the flavonoids with variations from April to August for *E. acuminatum*, *E. myrianthum*, *E. koreanum*, and *E. wushanense* [18,19,20,21]. Our study showed that the content of total flavonoids was much higher in April than that in August, while polysaccharide was much higher in August (Figure 1 and Figure 2). Thus, the combination of the major targeted compounds, different species and biomass yield should be considered when deciding the harvesting time. Additionally, the regional trial over 3 years indicated that the content of bioactive components of major flavonoids achieved relatively stability in the third year after transplantation (Figure 4 and Figure 5), which was different to *E. koreanum* for which a conclusion suggesting harvesting in the second year was reached [22]. This study provides valuable baseline data to formulate the better harvest time for *E. sagittatum* in artificial cultivation.

*E. sagittatum* is widely distributed in Central, Eastern and Southern China, and the results of much higher levels of flavonoid production in Central China followed by Northern then Southern China (Figure 6) is in agreement with its main distribution in Central China, which is also supported by the similar changing patterns of icariin and epimedin C in different years among the three regions (Figure 4). However, the result of higher contents in Beijing than in Guangzhou showed a discrepancy with the distribution of LT herbal epimedium accessions to other populations of *E. sagittatum* [8], although a significant correlation was found between the content of main flavones and the geographic location. Further studies are urgently needed to formulate a good agricultural practice for *E. sagittatum* cultivation.

## 4. Experimental

### 4.1. Plant Materials and Experimental Designs

**Light-controlled indoor cultivation**: Clonally propagated LT plants from an original 2007 introduction in Wuhan Botanical Garden were planted in pots in the end of February 2011 during dormancy, with 15 plants in each of three incubators and the same soil formula of mixture of 20% sand, 30% humus and 50% garden soil. Different temperatures at 15 °C, 20 °C, 25 °C, 28 °C, and 30 °C were set for entire growing season during April to July, with humidity maintained at RH 70%. According to the shade-tolerant character and the light intensity of cultivation outdoor, three light intensities were set including 20–35 μmol/m^2^/s, 40–100 μmol/m^2^/s and 160–350 μmol/m^2^/s, which were measured by a LI-6400XT system (Li-COR, Lincoln, NE, USA). Every plants plants were set as a duplicate and the middle leaflet of the ternate leafs was collected, respectively, at full-bloom stage (on April 20) and fructification stage (on August 20), cleaned and dried in shade for later analysis.

**Common-garden cultivation under different light intensity**: Plants from a wild *E. sagittatum* population collected from Luotian County in Hubei Province were arranged in two plots at the Wuhan Botanical Garden in 2007. The distance of the two plots was about 1,000 meters, and plot 1 was under artificial shade, while plot 2 was under natural woods of pines and firs. Both plots used the same soil as above. The environmental factors of the two plots were mainly different in light intensity, with 60–160 μmol/m^2^/s in plot 1 and 200–650 μmol/m^2^/s in plot 2 respectively, measured on sunny days at about 10 am in June. A total of 15 plants were randomly selected with every three plants set as a duplicate and sampled as above in 2011.

**Regional trial in different regions of China:** The same plant materials described above were transplanted in 2007 in the Vegetable Research Center, Beijing Academy of Agriculture and Forestry Sciences (Beijing, Northern China, 116.46°E, 39.92°N), Wuhan Botanical Garden of the Chinese Academy of Sciences (Wuhan, Central China, 114.36°E, 30.54°N, plot 1), and South China Botanical Garden of the Chinese Academy of Sciences (Guangzhou, Southern China, 113.14°E, 23.10°N), respectively. All locations used artificial shading with a single layer of sunshade net with the same light conditions (Beijing 50–180 μmol/m^2^/s, Wuhan 60–160 μmol/m^2^/s, Guangzhou 55–170 μmol/m^2^/s) and the same soil as above. A total of 15 plants at the full-blooming stage (in April) were randomly selected and analyzed for four consecutive years (from 2008 to 2011, among which, the results in 2010 was not used because of the bad storage of samples).

### 4.2. Chemicals and Reagents

Standards (99% purification) of epimedin A, epimedin B, epimedin C and icariin were purchased from Pulse Biological Technology Co., Ltd (Chengdu, China). Water was purified using a Milli-Q water system (Millipore; Bedford, MA, USA). Other reagents were analytical grade, including anhydrous glucose, phenol, ethanol, methanol, acetonitrile, acetic acid and concentrated sulfuric acid (Sinopharm Chemical Reagent Co, Ltd., Shanghai, China).

### 4.3. Total Flavonoid Content Analysis

An improved UV-spectrophotometry method was used [23]. Leaf samples were prepared suing No. 6 pharmacopoeia sieve, 0.250 mm mesh and 10 mg samples were added to 50% ethanol solution (30 mL) and then extracted with the aid of ultrasonic waves at 50 °C for 3 × 30 min. Consequently, the testing solution was obtained from the extract filtered through a 0.22 μm microfiltration membrane with 5–10 fold dilution. Furthermore, five concentrations of 9, 15, 20, 29 and 40 mg/L were prepared with standard epimedin C, and the regression equation of the standard curve y = 25.229x − 0.0811 and R^2^ = 0.9965 was calculated with RSD lower than 3%, accuracy of 98.8%–106.7%, LOD of 0.38 mg/L and LOQ of 1.21 mg/L. The determination wavelength was 270 nm. A U-3900UV-VIS spectrophotometer (Hitachi High-Technologies Corporation, Tokyo, Japan) was used.

### 4.4. Polysaccharide Content Analysis

An improved phenol-sulphuric acid method was used [23]. A total 0.5 g sample was added to 95% ethanol solution (50 mL), and extracted under water bath heating for 2 h before being filtered through filter paper. Then, the residue was washed with ethanol solution three times and water added at 25:1 (mL/g) liquid-solid ratio before extraction under ultrasonic waves for 3 × 30 min at 55 °C. After that the extract was centrifuged at 3,250 g for 5 min and the supernatant was collected together in the 250 mL flask after cooling and made up to volume with water. Furthermore, anhydrous glucose (25 mg) dried at 105 °C were weighed out to make a glucose mother solution of 0.1 mg/mL, from which the testing glucose solutions of 1, 2, 8, 14, 16 and 20 mg/L were prepared, adopting the homogeneous solution of 4.0 mL water + 1.0 mL of 5% phenol solution + 5 mL concentrated sulfuric acid as blank solution. The regression equation of y = 0.0122X + 0.0353, R^2^ = 0.9919 was draw with RSD less than 3%, accuracy of 95.0%–101.4%. LOD of 0.11 mg/L, LOQ of 0.23 mg/L, Then, all the standards and samples were tested using the U-3900UV-VIS spectrophotometer. The determination wavelength was 490 nm.

### 4.5. Analysis of Icariin, Epimedin A, Epimedin B and Epimedin C Contents

The determination method followed Gao [24]. After being heated for 4 hours at 60 °C, ground and sifted through No.6 pharmacopoeia sieve (0.250 mm mesh), a sample (50 mg) was weighed and added to 70% ethanol solution (5 mL) and extracted under ultrasonic waves for 30 min, then the solution for HPLC analysis was obtained from the extract by filtering through a 0.22 μm microfiltration membrane. Eluent A contained acetonitrile and eluent B consisted of 36% acetic acid and water (4:100 v/v). The gradient elution program was as follows: 0–15 min (22%–28% A), 15–25 min (28%–35% A), 25–40 min (35%–40% A), and 40–50 min (40%–45% A). Column was washed with 100% eluent A between every two testing samples for 10 min, and then re-equilibrated with 20% eluent A for 6 min. During the elution process, the flow rate remained at 1.0 mL/min with column temperature at 25 °C, detection wavelength of 272 nm and injection volume of 5 μL. Furthermore, mother solution obtained from 1.4 mg standard epimedin A, 2.0 mg standard epimedin B, 4.8 mg standard epimedin C, and 4.8 mg standard icariin weighed and dissolved in 1 mL methanol and seven gradient concentrations of each with epimedin A (14–350 ng/μL), epimedin B (20–500 ng/μL), epimedin C (48–1,200 ng/μL) and icariin (48–1,200 ng/μL) were prepared. The linearity of the concentrations of each flavonoid was tested with RSD less than 2.85% and the each recovery rate was 100.7%, 104.8%, 103.8% and 104%, respectively A Zorbax SB-C18 column was used as the chromatographic column for chromatographic analysis (250 mm × 4.6 mm I.D., 5μm) (Agilent Technologies, Palo Alto, CA, USA).

### 4.6. Data Analysis

Data of HPLC processing was done by Agilent ChemStation software, version A.10.02. ANOVA analysis of all the data were carried out with SPSS16.

## 5. Conclusions

The variations of medicinal components in LT herbal epimedium geographical accession were investigated under different light intensities and in different geographic regions. Icariin was the highest-content medicinal component among the four main flavones. The light intensity of 40–160 μmol/m^2^/s was recommended for cultivation practice for high production of total flavonoids. The polysaccharide accumulation was negatively correlated with light intensity. Central and Northern China were the most suitable regions for cultivation of the LT accession, which could be harvested at large scale in the third year after transplantation. There was a metabolic balance between flavonoids and polysaccharide at different growth stages, so the best harvesting stage should be considered comprehensively considering the combination of the major target compounds, different species, and the total biomass. 

## Figures and Tables

**Table 1:** ANOVA analysis of total flavonoids and polysaccharide under different cultivation conditions (common-garden and light-controlled indoor).

Source	*df*	MS	*F*	*P*
Polysaccharide (April)	4	0.17	294.21	<0.001
Polysaccharide (August)	4	0.37	1409.00	<0.001
Flavone (April)	4	0.10	2969.00	<0.001
Flavone(August)	4	0.00	12.41	<0.001

**Table 2:** ANOVA analysis of flavonoid contents under two light intensities (60–160 μmol/m^2^/s, plot 1; 200–650 μmol/m^2^/s, plot 2) in Wuhan Botanical Garden.

Source	*df*	MS	*F*	*P*
Epimedin A	1	0.62	1398	<0.001
Epimedin B	1	0.8	1124	<0.001
Epimedin C	1	0.16	206.4	<0.001
Icariin	1	0.01	29.4	0.001
Total	1	0.01	28.8	0.001

**Table 3:** ANOVA analysis of effects of cultivation duration and location on contents of four flavonoids.

		Epimedin A		Epimedin B		Epimedin C		Icariin		Total	
Source	df	MS	*F*	MS	*F*	MS	*F*	MS	*F*	MS	*F*
Duration (D)	2	4.57	218.46 **	568.76	28.32 **	1.12	44.09 **	6.97	95.41	2.35	189.19 **
Location (L)	2	0.53	25.52 **	571.77	28.47 **	1.00	39.63 **	0.34	4.70	0.66	52.62 **
D × L	4	0.04	1.94	275.76	13.73 **	0.41	16.12 **	0.70	9.527	0.19	14.97 **

Note: ** *p* < 0.001.
